# Graphene-based biosensors for the detection of prostate cancer protein biomarkers: a review

**DOI:** 10.1186/s13065-019-0611-x

**Published:** 2019-09-03

**Authors:** Li Xu, Yanli Wen, Santosh Pandit, Venkata R. S. S. Mokkapati, Ivan Mijakovic, Yan Li, Min Ding, Shuzhen Ren, Wen Li, Gang Liu

**Affiliations:** 1 Laboratory of Biometrory, Division of Chemistry and Ionizing Radiation Measurement Technology, Shanghai Institute of Measurement and Testing Technology, Shanghai, 201203 People’s Republic of China; 2 Division of Systems and Synthetic Biology, Department of Biology and Biological Engineering, Chalmers University of Technology, 41126 Gothenburg, Sweden; 3 The Novo Nordisk Foundation Center for Biosustainability, Technical University of Denmark, 2800 Lyngby, Denmark

**Keywords:** Prostate cancer, Protein biomarker, Graphene, Biosensor

## Abstract

Prostate cancer (PC) is the sixth most common cancer type in the world, which causes approximately 10% of total cancer fatalities. The detection of protein biomarkers in body fluids is the key topic for the diagnosis and prognosis of PC. Highly sensitive screening of PC is the most effective approach for reducing mortality. Thus, there are a growing number of literature that recognizes the importance of new technologies for early diagnosis of PC. Graphene is playing an important role in the biosensor field with remarkable physical, optical, electrochemical and magnetic properties. Many recent studies demonstrated the potential of graphene materials for sensitive detection of protein biomarkers. In this review, the graphene-based biosensors toward PC analysis are mainly discussed in two groups: Firstly, novel biosensor interfaces were constructed through the modification of graphene materials onto sensor surfaces. Secondly, ingenious signal amplification strategies were developed using graphene materials as catalysts or carriers. Graphene-based biosensors have exhibited remarkable performance with high sensitivities, wide detection ranges, and long-term stabilities.

## Introduction

PC is one of the most common cancers in the world which causes a fatality of approximately 10% in all cancer patients [1–4]. PC is a type of malignant neoplasm of the prostate gland which is extremely prevalent among men of age 50 and older [5, 6]. The established risk factors for PC include advancing age, race, positive family history of PC and diet [7, 8]. Being asymptomatic, it is very difficult to detect PC at early stages [9]. In clinical practice, early screening and diagnosis of PC is the most effective approach for reducing mortality [9, 10]. Thus, there is a growing body of literature that recognizes the importance of new technologies for early screening and diagnosis of PC [11, 12].

Tumor markers for early clinical screening and rapid diagnosis cover a wide range of biochemical entities, including, proteins [13, 14], nucleic acids [15–17], small metabolites [18, 19], cytogenetic and cytokinetic parameters [20], and entire tumor cells [21, 22] in body fluid [23]. So far, protein biomarkers are still recognized as a golden standard for PC diagnosis [24]. In the past few decades, a variety of promising biosensors have been developed based on the specific recognition of PC protein biomarkers, aiming at better performance of cancer diagnosis such as easy operation, portability, and real-time analysis [25–28]. Among them, the graphene-based biosensors have received considerable critical attention for the potential use in point-to-care (POC) testing devices, because of the unique properties of graphene such as large surface area, high electrical conductivity, excellent biocompatibility and convenient production/functionalization [29–31].

This review highlights recent graphene-based biosensors for PC protein biomarkers detection. As far as we know, this is the first review that focuses on specific one disease. We reviewed recent progress of graphene-based biosensors for PC protein biomarker detection. Our manuscript clearly stated the advantages and shortcomings of most of the graphene-based when facing PC diagnosis, thus, the manuscript should be valuable for the future application of graphene-based biosensors.

## Most commonly used protein biomarkers for PC detection

Protein biomarkers for cancer diagnosis are usually produced by either cancer cells or other cells in response to cancer [32–34], which have been proved to be promising targets for early diagnosis, monitoring treatment response, detecting recurrence or following up prognosis of cancer [35–37]. Protein biomarkers are usually in low abundance and unstable in body fluids, and thus, the specific detection of protein biomarkers is usually affected by the crude or complex environment [33, 38]. Thus, sensitivity, specificity, and accuracy are basic requirements to consider for protein biosensor fabrication [39–41].

Prostate-specific antigen (PSA) [42], which is also called human kallikrein 3 (hK3 or KLK3), has been widely recognized in clinical application as one of the earliest found, serological PC biomarkers [43, 44]. The PSA value above 4.0 ng/mL is usually considered as abnormal [45], thus, 4.0 ng/mL of PSA is the internationally recognized threshold value for PC occurrence [46, 47]. However, the specificity of PSA is still limited [48], because higher PSA levels can also be found in benign conditions, such as benign prostatic hyperplasia (BPH) [49–51], and PSA could be produced by normal breast and breast cancer cells [48]. These limitations indicate that PSA alone is not an appropriate surrogate marker for the diagnosis and screening of PC. Fortunately, several other protein PC biomarkers are developed.

Prostate-specific membrane antigen (PSMA) [52] is a type II transmembrane protein, and PSMA expression has been reported in benign prostatic hyperplasia and increased to higher lever in high-grade prostatic intraepithelial neoplasia and prostatic adenocarcinoma [53]. Further, stronger PSMA expression correlates to malignancy [54, 55]. The available research results suggest the potential clinical use for PSMA in PC patients. So far, the major PSMA clinical application has been in therapeutics and imaging [56–58]. Prostate stem cell antigen [59] is another recently discovered PC biomarker [60], which is highly expressed by a large number of human prostate tumors, such as metastatic and hormone-refractory, but barely expressed in normal tissues [60–62]. Engrailed-2 (EN2) protein is found in the urine sample of prostatic cancer patients and showed a specificity of 88.2% and a sensitivity of 66% [63, 64]. Therefore, the EN2 in urine is widely recognized as a potential biomarker of PC.

## Properties of graphene materials in biosensor study

Graphene is a two-dimensional (2D) nanomaterial, which plays an important role in the biosensor field [64–66]. The use of graphene in biosensing platform offers remarkable physical, optical, electrochemical and magnetic properties [67–70]. Different kinds of graphene materials are researched in biosensors including pristine graphene and functionalized graphene such as graphene oxide (GO), reduced graphene oxide (rGO), and graphene-based quantum dots (GQDs), etc. [71–74]. Pristine graphene is identified as the array of a 2D hexagonal lattice of sp^2^-bonded carbon atoms. GO is chemically produced by oxidation and exfoliation of graphene, causing extensive oxidative modification of the basal plane [31, 75–77]. The rGO is prepared through reductive process of GO, for this purpose, different methods have been developed to reduce its oxygen content, including thermal, chemical, microwave, photochemical, microbial/bacterial, and photo-thermal methods [78–80]. GQDs consist of single to tens of layers of graphene with a size of a few nanometers which exhibit quantum phenomena [81, 82].

**Fig. 1:**
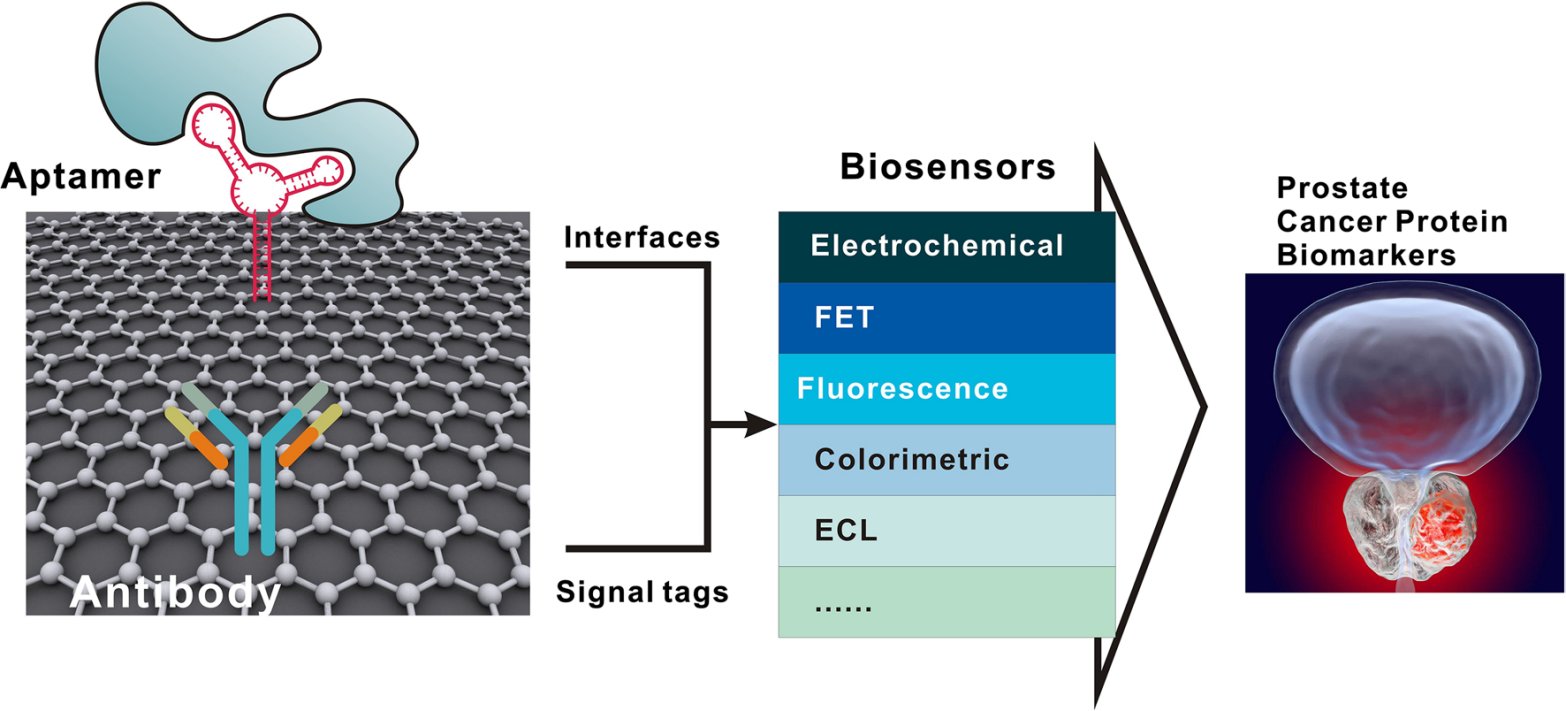
For prostate cancer detection, graphene materials are applied for the construction of novel interfaces and signal tags, on different analysis platforms including electrochemical, FET, fluorescence, colorimetric, ECL biosensors, et al

Development of protein biosensors based on graphene could be classified into two main groups (Fig. 1): Firstly, functioned graphene materials including GO, rGO and GQDs [72] were assembled onto the biosensor surface [electrode, field-effect transistors (FET) channel, etc.] to construct novel biosensor interfaces for improved assembling of molecular receptors [83]. In this group, excellent biosensor performance was achieved mainly based on the increased specific surface area and the unique π–π orbital interaction on the interface. Secondly, many recent studies applied graphene materials as excellent carriers for the construction of novel nanocomposites [84], and in this group, interesting biosensor signal amplification and unique catalytic/chemical activity was realized for sensitive protein biomarker analysis [85].

## Biosensor interfaces based on graphene

Graphene and its derivatives are studied for the construction of novel biosensor interface [67], which is critical for interface-based biosensors including electrochemical biosensors, electrochemiluminescent (ECL) biosensors and FET biosensor [86]. Many recent studies reported that nanocomposites based on graphene showed improved capability of combining different biomolecules, with higher surface area [87] and excellent biocompatibility [88].

### Construction of antibody-graphene biosensor interface

Traditionally, antibodies are physically adsorbed onto the immune-assay surfaces, such as classic 96-well plates and colloidal gold test strips. However, one of the main obstacles is the affinity and capacity, because the hydrophobic and hydrophilic interaction is relatively weak and the orientation of the antibody molecules is random [89]. As several recent studies reported, the strong cross-linking between carboxylic acid groups on graphene materials and the amine groups of antibodies (COOH-NH_2_) was used for the assembling of antibody on novel biosensor interfaces [90, 91]. In their work, the application of graphene materials increased the loading amount, orientation controllability as well as binding capability of the antibodies or antibody fragments. For example, Li et al. developed a graphene modified sensor platform with increased surface area, and then assembled antibody onto the surface through COOH–NH_2_ combining, with the assistant of 1-ethyl-3-(3-dimethylaminopropyl) carbodiimide (EDC) and *N*-hydroxysuccinimide (NHS), and they finally achieved a low detection limit of 2 pg/mL [92].

**Fig. 2:**
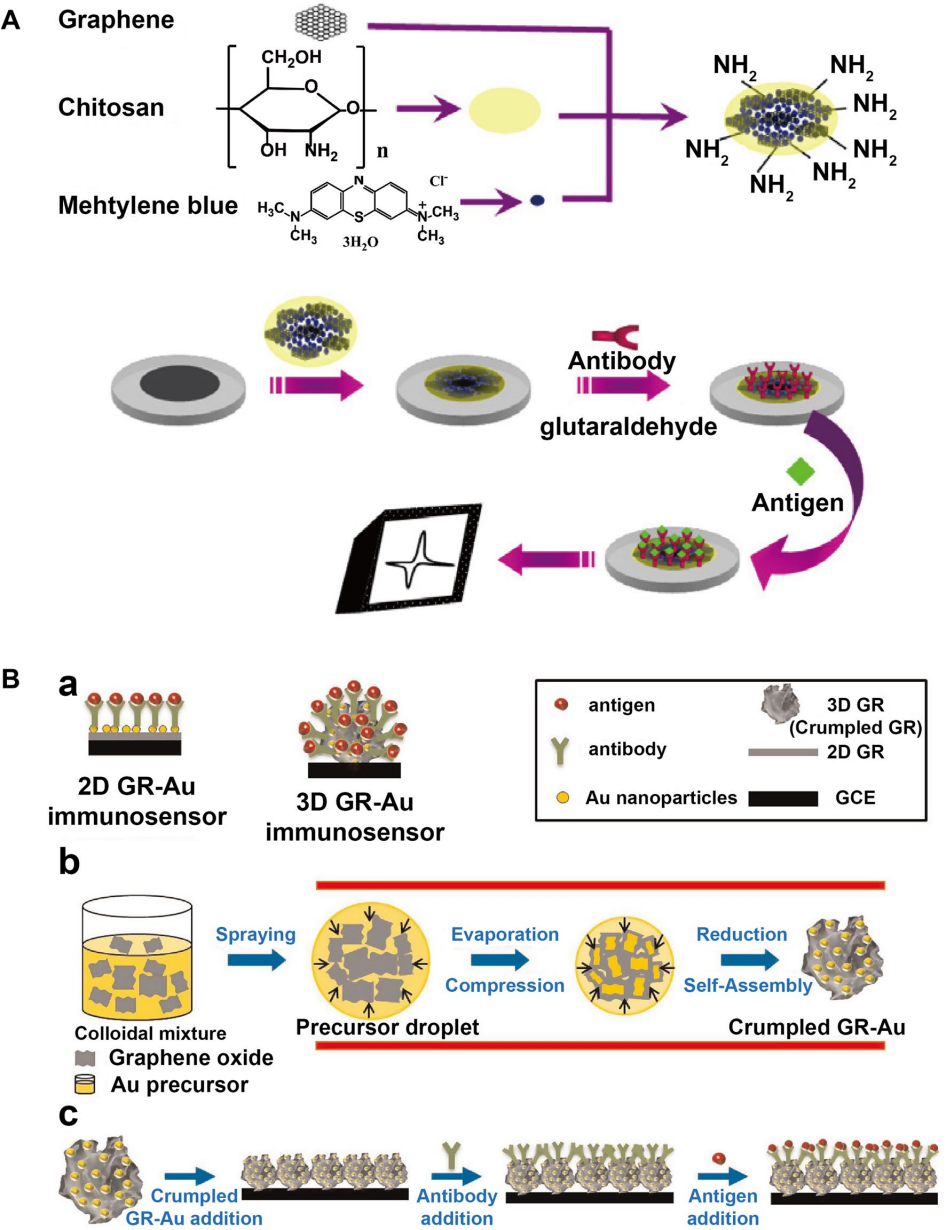
Schematic illustration of Label-free electrochemical immunosensors for PC protein biomarkers based on: **A** graphene-methylene blue nanocomposite, Reprinted with permission from [93], Copyright 2011 Elsevier. **B** graphene-Au nanocomposite (Reprinted with permission from [94]. Copyright 2014 Elsevier)

In order to realize better-oriented assembling of antibody, Mao et al. applied chitosan as the dispersant to construct an immuno-interface on a glassy carbon electrode (Fig. 2A), which provided much more amino groups for PSA antibody bonding. They finally developed a simple, label-free electrochemical immunosensor on graphene-methylene blue composite modified electrode [93]. More recently, Jang et al. developed a novel 3D graphene-Au composite (Fig. 2B), toward increased accessible surface area for antibody combination than 2D graphene sheet. More importantly, the crumpled graphene could produce higher capacitances, which is crucial for the following electrochemical immunosensing [94].

**Fig. 3:**
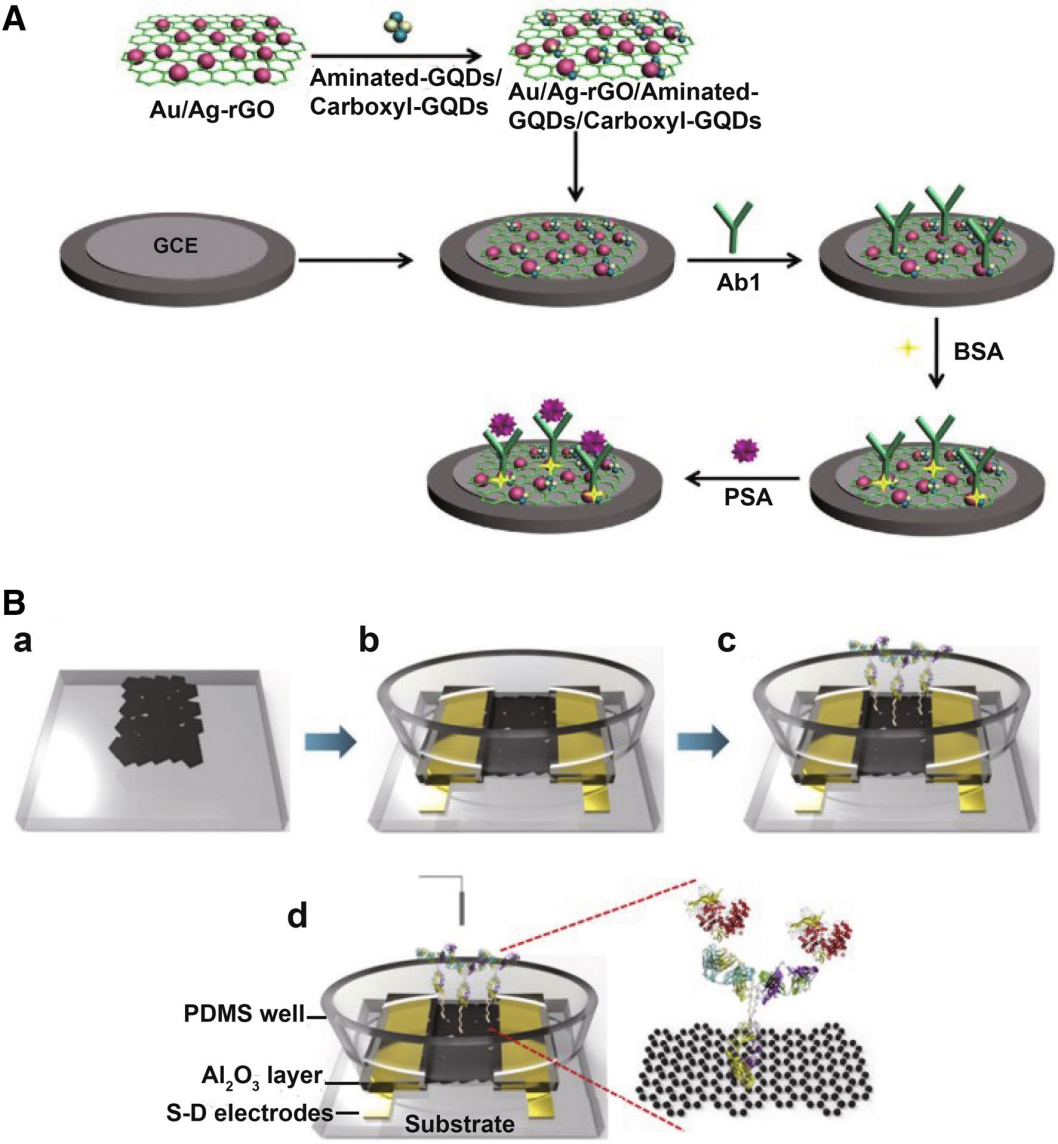
Illustration of PSA immunosensor fabrication process. **A** An ECL immunosensor on the electrode surface modified with Au/Ag-rGO, Reprinted with permission from [96]. **B** FET immunosensor on an rGO channel (Reprinted with permission from [105]. Copyright 2012 Elsevier)

A graphene-modified electrode was also reported in ECL biosensor [95] for PSA detection. More recently, Wu et al. developed an electrode surface modified with Au/Ag–rGO (Fig. 3A), and then, a large amount of aminated GQDs and carboxyl GQDs were combined onto the electrode surface. In their work, Au and Ag nanoparticles were used for the adsorption of PSA antibody, and meanwhile, GQDs were for the ECL signal amplification. Finally, they constructed a label-free PSA ECL biosensor with a detection limit as low as 0.29 pg/mL [96].

Graphene materials were also applied in FET biosensors, for the construction of 2D nano-FET biosensors [97–101], with unique advantages like more receptor biomolecules, low noise, and high sensitivity, compared with 1D FET biosensors [102–104]. As a successful example, Kim and coworkers [105] developed an rGO-based FET biosensor for label-free and ultrasensitive analysis of PSA/α1-antichymotrypsin (PSA-ACT) (Fig. 3B). The FET biosensor was produced by combining rGO onto an aminated glass surface, and then, functionalized with PSA antibody. When PSA-ACT was captured by the antibodies on FET substrate, a linear shift of the gate voltage (Δ*V*_*g*,*min*_) was achieved, indicating the minimum conductivity. Finally, they successfully performed detection of PSA-ACT of femtomolar level.

### Construction of aptamer-graphene biosensor interface

For interface-based PC biosensors, the DNA capture probe plays a key role, which could recognize and capture the target molecules [106]. The very famous DNA probe in PC biosensor is DNA aptamer [10, 107–109], which is a special single-strand DNA (ssDNA) isolated from DNA/RNA libraries of random sequence, by using an in vitro selection process called systematic evolution of ligands by exponential enrichment (SELEX) [110–113].

As the first step toward an aptasensing platform, scientists developed several different strategies to assemble the DNA aptamer onto the electrode as the key recognition element [114–117]. In many reported studies, graphene-based nanocomposites were firstly prepared consisting of graphene and another combing material. For example, Bafrooei et al. modified the electrode with rGOmulti-walled carbon nanotube (MWCNT) nanocomposite and then produced a layer of gold nanoparticles (AuNPs) through electrochemical reduction under − 0.2 V in HAuCl_4_, then SH-labeled DNA aptamer was combined to Au on the electrode surface. Finally, their aptasensor achieved 1.0 pg/mL limit of detection (LOD) by using both DPV and ESI methods. Different chemical reactions were applied for the assembling of DNA onto graphene materials. Branched polyethylenimine (PEI) was applied by Pan et al. to connect thiol-mediated ssDNA onto carboxylated GO for PSA detection [118]. Recently, EDC-NHS coupling was applied by Settu et al. to combine DNA probe onto a screen-printed carbon-graphene-modified electrode of the detection of EN2 protein [119].

## Graphene-based composites for signal-amplification

### Peroxidase-like activity of GO

**Fig. 4:**
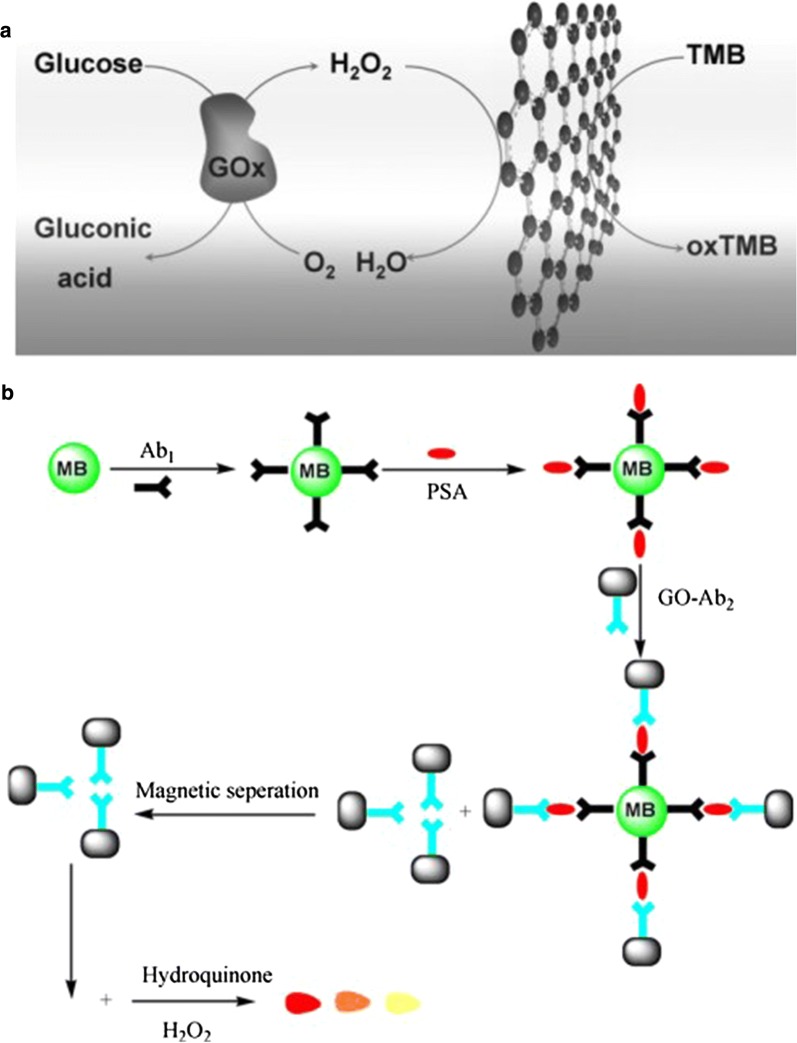
**a** Schematic illustration of peroxidase-like activity of GO for the colorimetric detection of glucose, Reprinted with permission from [120], Copyright 2010, John Wiley and Sons, **b** schematic representation of the immunoassay procedure (Reprinted with permission from [134]. Copyright 2010 Elsevier)

In 2010, Qu’s group firstly reported the peroxidase-like activity of GO (Fig. 4a) [120]. Before long, Yang and coworkers found GO was capable of catalyzing the oxidation of hydroquinone with the assistant of H_2_O_2_, producing a brown color solution. Thus, they produced an antibody-functionalized GO as the signal tag and developed a sandwich-type colorimetric immunoassay for the detection of PSA. In their work [121], an immunocomplex was established when PSA combined GO with secondary anti-PSA (GO-Ab_2_) and magnetic bead (MB) with primary anti-PSA antibody (MB-Ab_1_). After the separation in a magnetic field, the color signal was detected corresponding to the concentration of PSA. Their simple immunoassay can be detected by naked eyes (Fig. 4b).

### Graphene materials being applied as the carrier of signal tags

Many recent studies applied graphene-related materials as excellent carriers for the construction of novel nanocomposites for biosensor signal amplification [122–124]. These graphene-based composites were developed by combining graphene or its derivates with metal oxides, metal nanoparticles, or conductive polymers, etc., and this kind of composites showed unique catalytic/chemical activity [86], that has been widely applied in PC biosensors [125].

Han et al. developed a novel signal tag for PSA and free PSA (fPSA) detection, by using onion-like mesoporous graphene sheets (O-GS) as the carrier of different AuNP-based nanohybrids [126]. As the novel O-GS have multilayer lamellar structure, large surface-to-volume ratio, and excellent electronic transport properties, two kinds of redox nanocomposites were attached to the surface of O-GS, which could accelerate the electron transfer rate and enhance the immobilization amount of enzyme and detection antibodies. Sun et al. reported a signal label by combining bovine serum albumin (BSA)-stabilized silver nanoparticles onto ZnO nanorods modified rGO, and the AgNPs in the composite showed super catalytic performance toward hydrogen peroxide (H_2_O_2_), generating a current signal [127]. Feng et al. developed a sandwich-type electrochemical immunosensor for the detection of PSA. In their work, a GO platform (Au@Th/GO) was used to immobilize primary antibodies and accelerate the electron transfer on the electrode interface. An rGO-based nanocomposite (PtCu@rGO/g–C_3_N_4_) with large surface area, good biocompatibility, and excellent conductivity were used as labels for combining secondary antibodies and amplifying signals. Then secondary antibodies were combined onto this platform and signals were amplified from H_2_O_2_ reduction [128].

**Fig. 5:**
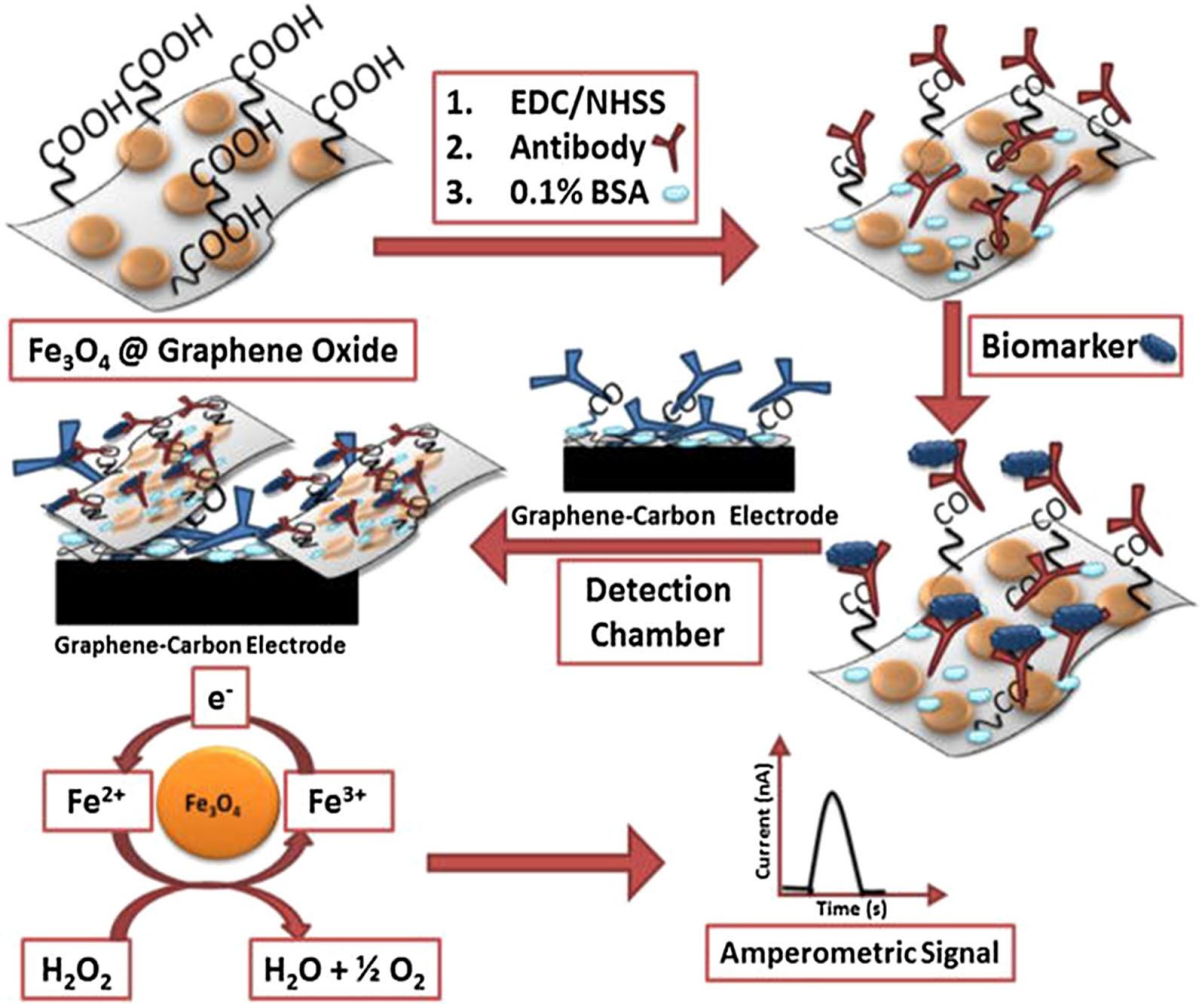
Protein capture and detection mediated by Fe_3_O_4_@GO sheets. Proteins captured by Fe_3_O_4_@GO decorated with detection antibodies. Composite with biomarker was then captured on the sensor surfaces coated with graphene and capture antibodies. Amperometric signal was generated by injecting 100 μL 5 mM H_2_O_2_ (Reprinted with permission from [129]. Copyright 2016 Elsevier)

Sharafeldin et al. [129] assembled Fe_3_O_4_ nanoparticles together with antibody onto GO sheets to produce a multi-function nanocomposite (Fig. 5). When the GO-antibody-Fe_3_O_4_ nanocomposite specifically combined to PSA and PSMA proteins, the resulted complex could be isolated in a magnetic field and delivered in microfluidic channel to an electrochemical detection cell. The Fe_3_O_4_–GO particles subsequently catalyze H_2_O_2_ reduction, generating a current signal. Improved LOD of 15 fg/mL of PSA and 4.8 fg/mL of PSMA was achieved, which was 1000-times better than previously reported PSA biosensors using Fe_3_O_4_ only, probably because GO carried more Fe_3_O_4_ particles and thus dramatically increased the electrochemical signal.

## Conclusion and future perspectives

Biosensors for cancer biomarker detection opened a new avenue for the POC PC detection. In spite of their very short history, graphene-based materials have successfully demonstrated their unique advantages in biosensors for PC protein biomarkers. This review has summarized recent advances, challenges, and trends in the application of graphene-based materials for biosensing of PC protein biomarkers. In this review, the commonly used PC protein biomarkers for biosensor, the unique properties of graphene and the roles of graphene-based materials for biosensing were introduced. Among various PC protein biomarkers, PSA was the most frequently selected target for PC detection biosensor construction. Most studies focused on single biomarker detection and studies on detection of multiple biomarkers are limited. A variety of graphene-based materials such as pristine graphene, functionalized graphene (GO, rGO, GODs) were used in PC biosensor development and most of them were combined with other nanomaterials like nanoparticles. We have also summarized various strategies and approaches which can be used for graphene-based biosensor development. Graphene-based materials were used not only for novel biosensor interfaces construction but also as excellent carriers for the construction of novel nanocomposites for signal amplification. In most of the cases, graphene-based biosensors have exhibited satisfactory biocompatibility towards the bioactive species and remarkable performance with high sensitivities, wide linear detection ranges, low detection limits and long-term stabilities (Table 1). As other 2D materials have now been explored, we believe that more 2D materials like MoS_2_ could be employed and integrated into biosensors for PC biomarker detection in the upcoming future.

**Table 1:** Current generation reports of graphene-based biosensors for PC biomarker detection

Technique	Receptor system	Target proteins	LOD	Detection ranges	References
ECHEM	rGO-MWCNT/AuNPs	PSA	1.0 pg/mL	(0.005–20) ng/mL for DPV, (0.005-100) ng/mL for EIS	[132]
ECHEM	rGO/Ag@BSA	HCG, PSA, CEA	0.0007 mIU/mL for HCG, 0.35 pg/mL for PSA, and 0.33 pg/mL for CEA	(0.002-120) mIU/mL for HCG, (0.001–110) ng/mL for PSA, (0.001–100) ng/mL for CEA	[127]
ECHEM	Au@Th/GO, PtCu@rGO/graphitic carbon nitride	PSA	16.6 fg/mL	50 fg/mL–40 ng/mL	[128]
ECHEM	GO/ssDNA/PLLA NPs	VEGF, PSA	–	(0.05-100) ng/mL for VEGF, (1-100) ng/mL for PSA	[118]
ECHEM	Fe_3_O_4_/PDDA/GO	PSA, PSMA	15 fg/mL for PSA, 4.8 fg/mL for PSMA	(61 fg/mL–3.9 pg/mL) for PSA, (9.8 fg/mL–10 pg/mL) for PSMA	[129]
ECHEM	Au@PBNPs/O-GS, Au@NiNPs/O-GS	fPSA, PSA	6.7 pg/mL for fPSA, 3.4 pg/mL for PSA	(0.02–10) ng/mL for fPSA, (0.01–50) ng/mL for PSA	[126]
ECHEM	GS/DA/Fe_3_O_4_/FC	PSA	2 pg/mL	(0.01–40) ng/mL	[92]
ECHEM	Carbon-graphene/aptamer	EN2 protein	38.5 nM	(35–185) nM	[119]
ECHEM	GS-MB-CS	PSA	13 pg/mL	(0.05–5.00) ng/mL	[93]
ECHEM	3D graphene/Au	PSA	0.59 ng/mL	(0–10) ng/mL	[94]
FET	rGO	PSA-ACT	100 fg/mL	(10^−7^–1) μg/mL	[105]
Fluorescence	GQDs–NR	ACP	28 μU/mL	(0–1500) μU/mL	[133]
Fluorescence	GO/peptide/FITC	PSA	0.3 nM	(0–20) nM	[64]
ECL	Au/Ag-rGO/aminated-GQDs/carboxyl-GQDs	PSA	0.29 pg/mL	1 pg/mL–10 ng/mL	[96]
ECL	graphene	PSA	8 pg/mL	10 pg/mL–8 ng/mL	[95]
Colorimetric	GO/MB	PSA	–	–	[134]

Although tremendous progress has been made in the past a few years of graphene-based biosensors for PC detection, there still remain some challenges. Firstly, PSA has been demonstrated not a specific biomarker in prostate cancer early screening. As a result, detection of multiple biomarkers is crucial for precise diagnosis and prognosis of PC [130, 131]. More attention should be paid to studies on the simultaneous detection of multiple biomarkers in the future. In addition, there are only a few studies on PC biomarker detection in different body fluid. To improve the accuracy and practicability of the diagnosis, more studies are expected to perform biomarker detection in different body fluid.

## Data Availability

Not applicable
